# Mycotic Brain Abscess Caused by Opportunistic Reptile Pathogen

**DOI:** 10.3201/eid1102.040915

**Published:** 2005-02

**Authors:** Christoph Steininger, Jan van Lunzen, Kathrin Tintelnot, Ingo Sobottka, Holger Rohde, Matthias Ansver Horstkotte, Hans-Jürgen Stellbrink

**Affiliations:** * University Clinic Eppendorf, Hamburg, Germany;; † Robert Koch-Institut, Mykologie, Berlin, Germany

**Keywords:** brain abscess, *Chrysosporium*, fungus, HIV infection, immunocompromised, letter

**To the Editor:** A 38-year-old, HIV-seropositive Nigerian man sought treatment with an 8-month history of severe parietal headache, impaired memory, fatigue, paresthesia of the left arm, and left-sided focal seizures. He had no history of neurologic disorders, including epilepsy. On physical examination, the patient appeared well, alert, and oriented, with slurred speech. Evaluation of the visual fields showed left homonymous hemianopsia. All other neurologic assessments were unremarkable. The patient had a blood pressure of 120/80, a pulse of 88 beats per minute, and a body temperature of 37.3°C. Leukocyte count was 8,600/µL, total lymphocyte count was 1,981/µL, CD4+ cell count was 102/µL, and CD4/CD8 ratio was 0.07. HIV RNA-load was <50 copies/mL; all other laboratory parameters were normal. The patient had received antiretroviral therapy (stavudine, lamivudine, nevirapine) for 5 months before admission, but no prophylaxis for opportunistic infections. Magnetic resonance imaging (MRI) of the brain disclosed 2 masses, 3.3 and 4.8 cm in diameter, respectively (Figure A), and signs of chronic sinusitis. A computed tomographic chest scan showed infiltration of both lower segments with multiple, small nodules (Figure B). Blood cultures were repeatedly negative. A computer-guided needle-aspiration of the brain lesions yielded yellow-brown, creamy fluid in which abundant septated fungal hyphae were detected microscopically (Figure C). Cytologic investigation was consistent with a necrotic abscess. The cycloheximide-resistant isolate was strongly keratinolytic and identified as a *Chrysosporium* anamorph of *Nannizziopsis vriesii* (*1**,**2*). High-dose antimicrobial treatment with voriconazole (200 mg twice daily, subsequently reduced to 200 mg daily) was added to the antiretroviral (ritonavir, amprenavir, trizivir), anticonvulsive, and adjuvant corticosteroid treatment. The isolate was highly susceptible to voriconazole in vitro (MIC, <16µg/mL [Etest, AB-Biodisk Solna, Sweden]). Recovery was complicated by a generalized seizure and severe, acute psychosis associated with rapid refilling of the 2 lesions with mycotic abscess fluid. After re-aspiration, the patient's psychosis improved gradually, and no further seizures occurred. When last seen 4 months later, the patient was healthy and without neurologic deficits. His CD4+ cell count was 233/µL, HIV-load was <50 copies/mL, and a MRI scan of the brain showed partial regression of the 2 brain lesions (Figure D).

**Figure:**
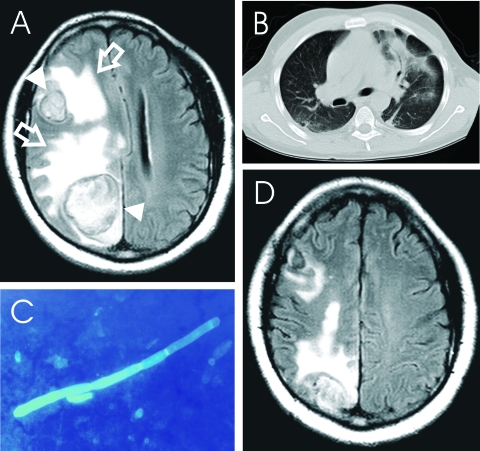
*Chrysosporium* sp. brain abscess in an HIV-seropositive patient. A) T2-weighted magnetic resonance imaging (MRI) scan of the brain showing 2 large masses (triangles) surrounded by a ring of signal intensity and extensive perifocal edema (open arrows), global swelling of the right hemisphere, and a midline shift of 1.2 cm. B) Computed tomographic scan of the chest showing infiltration of the left and right lower segment. C) Mold mycelium in aspirate of brain abscess with calcoflour white stain. D) T2-weighted MRI scan of the brain performed 4 months after beginning of therapy.

*Chrysosporium* spp. are common soil saprobes, occasionally isolated from human skin. Invasive infection is very rare in humans, and most were observed in immunocompromised patients, manifesting as osteomyelitis (*3**,**4*) or diffuse vascular brain invasion (*5*). Here, we report the first case of brain abscesses by the *Chrysosporium* anamorph of *N. vriesii.* This fungus has been associated with fatal mycosis in reptiles (*6**,**7*) and cutaneous mycosis in chameleons originating from Africa (*2*).

In our patient, we were unable to determine the portal of entry and the sequence of fungal dissemination; no skin lesions were present at the time of admission. However, the multifocal nature, lung infiltration, and involvement of the middle cerebral artery distribution suggest hematogenous dissemination (*8**,**9*) after replication of airborne conidia within the respiratory tract.

Fungi cause >90% of brain abscesses in immunocompromised transplant patients with an associated mortality rate of 97% (*10*), despite aggressive surgery and antifungal therapy (*9*). Our patient was treated successfully with abscess drainage, antiretroviral therapy, and oral voriconazole, a novel antifungal triazole drug. Despite limited data available on voriconazole penetration into brain abscess cavities (*9*), this drug was clinically and radiologically effective in our patient.
